# Risks of Decreased Renal Function and Increased Albuminuria for Glycemic Status and Metabolic Syndrome Components: Taichung Community Health Study

**DOI:** 10.1155/2014/841497

**Published:** 2014-05-12

**Authors:** Cheng-Chieh Lin, Chia-Ing Li, Chiu-Shong Liu, Wen-Yuan Lin, Chih-Hsueh Lin, Ming-May Lai, Yih-Dar Lee, Ching-Chu Chen, Chuan-Wei Yang, Tsai-Chung Li

**Affiliations:** ^1^Department of Family Medicine, China Medical University Hospital, Taichung 40402, Taiwan; ^2^School of Medicine, College of Medicine, China Medical University, Taichung 40402, Taiwan; ^3^Department of Medical Research, China Medical University Hospital, Taichung 40402, Taiwan; ^4^Medical College, Department of Psychiatry, Medical College, National Cheng Kung University, Tainan 70101, Taiwan; ^5^Bristol-Myers Squibb Ltd, Global Development & Medical Affair, Taipei 10586, Taiwan; ^6^Division of Endocrinology and Metabolism, Department of Medicine, China Medical University Hospital, Taichung 40402, Taiwan; ^7^Graduate Institute of Biostatistics, College of Management, China Medical University, Taichung 40402, Taiwan; ^8^Department of Healthcare Administration, College of Health Science, Asia University, Taichung 41354, Taiwan

## Abstract

*Background*. The objective of this study was to assess the association of glycemic status and decreased renal function as determined by estimated glomerular filtration rate (eGFR) and albuminuria in an adult Taiwanese metropolitan population. *Methods*. We did a cross-sectional survey in a representative sample of 2,350 Taiwanese adults aged 40 years and over living in a metropolitan city in Taiwan from 2004 to 2005. Glycemic status was classified as normal glycemia, hyperglycemia, and type 2 diabetes (T2D). Renal function was assessed with eGFR using modified Modification of Diet in Renal Disease Study equation for Chinese. Albuminuria was determined by the urinary albumin-creatinine ratio. Decreased renal function was defined as eGFR <60 mL/min/1.73 m^2^ and albuminuria as the albumin-creatinine ratio >30 mg g^−1^ creatinine. *Results*. 593 (25.23%) had hyperglycemia and 287 (12.21%) had T2D. As glycemia level increased, the prevalence of albuminuria and decreased eGFR increased. After adjustment, T2D was associated with an OR of 2.93 (95% CI: 2.11–4.07) for albuminuria, and an OR of 2.05 (95% CI: 1.18–3.58) for decreased eGFR. *Conclusions*. In a representative sample from a metropolitan city in Taiwan, T2D was associated with albuminuria and decreased eGFR.

## 1. Introduction

The growth of end-stage renal disease (ESRD) populations worldwide has been a concern for many countries. The prevalence and incidence of ESRD are also rapidly increasing in Taiwan. Using data from the US Renal Data System as a comparison, Taiwan has had the greatest incidence and the second greatest prevalence of ESRD since 2000 [1]. There were 2.6- and 3.7-fold increases in incidence and prevalence during the past decade in Taiwan. Thus, to identify significant predictors of renal function is important issue for Taiwan.

Based on the previous epidemiological study, the key predictors of chronic kidney disease in Taiwan are age, female sex, type 2 diabetes, hypertension, and hyperlipidemia [2]. Among these risk factors, type 2 diabetes, hypertension, and hyperlipidemia are components of metabolic syndrome. Particularly, the incidence of type 2 diabetes is rapidly increasing in Taiwan and has become the fourth major leading cause of death for men and women in Taiwan since 2002 [3]. As lifestyle behaviors have been westernized, the prevalence of type 2 diabetes has rapidly increased in Taiwan. According to the Nutrition and Health Survey in Taiwan, the prevalence of type 2 diabetes has increased from 0.6%, 11.4%, and 22% in females and 1.1%, 7%, and 7.2% in males in the population aged 19–44, 45–64, and ≥65 years, respectively, from 1993 to 1996, to 5.0%, 10%, and 24.5% in females and 5%, 15%, and 28.2% in males, respectively, from 2005 to 2008 [4, 5]. Although type 2 diabetes is the leading cause of ESRD, studies examining how glycemic status is related to albuminuria and decreased renal function expressed in terms of estimated glomerular filtration rate (eGFR) are limited [6–9]. In addition, most of these studies only considered renal dysfunction [6, 7, 9] and one study considered proteinuria determined by urine dipstick measurement or urine albumin-to-creatinine ratio (UACR) [8].

In clinical settings, the presence of albuminuria is essential for the diagnosis of early-stage renal dysfunction, and the status of albuminuria is not always accompanied with a decrease in GFR in patients with type 2 diabetes [10]. The GFR may still be normal during the initial positive testing phase for microalbuminuria, which is due, in part, to the early hyperfiltration phase associated with diabetes and obesity. Thus the relationships between glycemic status and decreased eGFR may be different from those between glycemic status and albuminuria. A cross-sectional study in the US identified a significant association between hyperglycemia and microalbuminuria but failed to detect an association between hyperglycemia and decreased eGFR [11]. To our knowledge, no study has reported the relationship of decreased eGFR and albuminuria simultaneously with glycemic status in a Taiwanese population. This cross-sectional survey was performed to examine the independent relationship between glycemic status and decreased renal function as determined by eGFR and albuminuria in a representative sample of the Taiwanese general population.

## 2. Subjects and Methods

### 2.1. Study Population

This was a cross-sectional epidemiological study based on data from the Taichung Community Health Study (TCHS). A total of 2,359 residents aged 40 and over in Taichung City, Taiwan, participated in October 2004. A two-stage sampling design was used, with a sampling rate proportional to size within each stage. At each stage, simple random sampling was used. A total of 4280 individuals were selected, and, of them, 750 were ineligible and were excluded, leaving 3,530 eligible subjects; 2,359 agreed to participate, with an overall response rate of 66.83%. The detailed methodology has been described elsewhere [12–15]. This study was approved by the Human Research Committee of China Medical University Hospital. Written informed consent was obtained from each participant.

### 2.2. Data Collection

Anthropometric measurements were obtained from the complete physical examination. Weight and height were measured on an autoanthropometer (super-view, HW-666), with the subjects shoeless and wearing light clothing. Body mass index (BMI) was derived from the formula of weight (kg)/(height)^2^ (m^2^). With the participant standing, waist circumference was measured midway between the superior iliac crest and the costal margin. Blood pressure was measured using an electronic device (COLIN, VP-1000, Japan).

Blood was drawn with minimal trauma from an antecubital vein in the morning, after a 12-hour overnight fasting, and was sent for analysis within four hours of collection. Biochemical markers such as fasting plasma glucose, HDL cholesterol (HDL-C), triglyceride, urine albumin, and creatinine were analyzed with a biochemical autoanalyzer (Beckman Coulter Synchron system, Lx-20, Fullerton, CA, USA) at the Clinical Laboratory Department of China Medical University Hospital. Plasma cholesterol and triglyceride levels were determined using an enzymatic colorimetric method. The HDL-C level was measured by a direct HDL-C method, and the low-density lipoprotein cholesterol (LDL-C) level was measured by a direct LDL-C method. UACR in the morning urine sample was used as a marker of the albumin excretion rate. Urinary creatinine (Jaffe's kinetic method) and albumin (colorimetry bromocresol purple) were measured with an autoanalyzer. The interassay precision coefficient of variation was <3.0% for both creatinine and albumin concentrations. UACR ranging over 30 mg g^−1^ creatinine was defined as albuminuria [16]. GFR was estimated on the basis of serum creatinine level, with the most recent expression of the Modification of Diet in Renal Disease Study (MDRD) prediction equation for standardized serum creatinine [16]. Specifically, eGFR (mL/min/1.73 m^2^) = 186.3 × (standardized serum creatinine in mg/dL)^−1.154^  × age^−0.203^ (× 0.742 if female) (× 1.2331 if ethnic Chinese) [17]. Decreased eGFR was defined as eGFR ≤ 60 mL/min/1.73 m^2^ [8], corresponding to stages 3 to 5.

The measurement of brachial-ankle pulse wave velocity (baPWV) and the ankle-brachial index (ABI) were determined using an automatic waveform analyzer (VP-1000; Colin Co., Komaki, Japan). Higher baPWV values indicated more severe arterial stiffness. Lower ABI values indicated more severe peripheral vascular disease (PVD). High baPWV was defined as a value higher than 1,400 cm/s, whereas an ABI index <0.9 was considered as the presence of PVD [18]. Using the Framingham risk score based on the LDL-C level [19], the estimated total coronary heart disease risk over a 10-year period for every individual was calculated. Data on sociodemographic characteristics, including gender, age, smoking, drinking, physical activity, occupational activity, menopausal status, family history of cardiovascular-related diseases, physician-diagnosed diseases, and medication history, were collected when the participants underwent a complete physical examination.

### 2.3. Glycemic Status and the Other Components of Metabolic Syndrome

Normal glycemia was defined as fasting glucose less than 100 mg/dL and no history of type 2 diabetes and hypoglycemic agent treatment or insulin treatment. Hyperglycemia was defined as elevated fasting glucose (≥100 mg/dL), whereas type 2 diabetes was defined as a fasting plasma glucose concentration ≥126 mg/dL and/or history of type 2 diabetes and hypoglycemic agent treatment or insulin treatment. The AHA/NHLBI statement was used to define the components of metabolic syndrome [20]: elevated waist circumference, elevated triglycerides (≥150 mg/dL), reduced HDL-C (<40 mg/dL for men, <50 mg/dL for women), and elevated blood pressure (≥130/≥85 mmHg). Elevated waist circumference was defined by Asia-Pacific cutoff limits: waist circumference of 90 cm or more for men and 80 cm or more for women, because of the importance of ethnic-specific cutoff points for waist circumference [21]. As stipulated by the definition, we included all individuals receiving pharmacologic treatment for hypertension as having elevated blood pressure and all subjects receiving fibrates as possessing both elevated triglycerides and reduced HDL-C.

### 2.4. Statistical Analysis

Decreased renal function was defined as eGFR <60 mL/minute/1.73 m^2^ based on clinical practice guideline [16]. Individuals with an eGFR below 15 mL/minute/1.73 m^2^ (*n* = 1) were excluded from all analyses. Continuous variables were reported as mean ± standard deviation (SD) and categorical variables were reported as percentages. Multivariate logistic regression models were fit to estimate the odds ratios of albuminuria and decreased eGFR for hyperglycemia and type 2 diabetes. In order to evaluate whether different cutoff points of eGFR and UACR have impact on our study findings, sensitivity analysis was performed by using cutoff point of 45 mL/min/1.73 m^2^ for eGFR and 17/25 (men/women) mg g^−1^ for UACR. All reported *P* values were those of two-sided tests; statistical significance was set at *P* < 0.05. All analyses were performed using SAS version 9.1 (SAS Institute Inc., Cary, NC).

## 3. Results

Table 1 shows the demographic and cardiovascular risk factors of study subjects according to renal dysfunction and albuminuria. Subjects with renal dysfunction had a higher mean age, male gender identity, a family history of diabetes, antihypertension medication use, cholesterol lowering medication use, peripheral vascular disease, arterial stiffness, albuminuria, higher Framingham risk scores, and metabolic syndrome components as well as metabolic syndrome. Similarly, subjects with albuminuria had a higher mean age, male gender identity, antihypertension medication use, cholesterol lowering medication use, arterial stiffness, higher Framingham risk scores, obesity, and metabolic syndrome components as well as metabolic syndrome.

**Figure 1:**
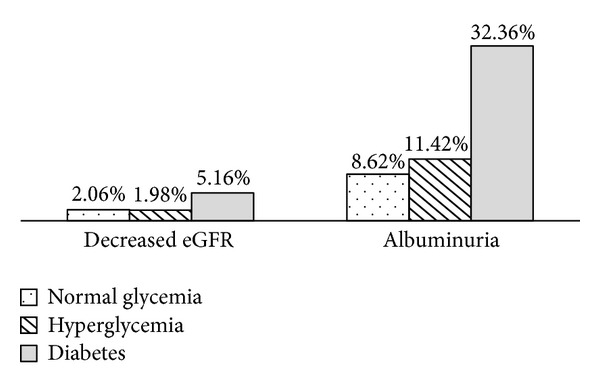
The age- and sex-adjusted prevalence of decreased eGFR and albuminuria in subjects with different glycemic statuses.

Figure 1 shows the prevalence of decreased eGFR and albuminuria in subjects with different glycemic statuses. The age- and sex-adjusted prevalence of decreased eGFR and albuminuria increased a little for individuals with hyperglycemia compared to individuals with normal glycemia but increased a lot for individuals with type 2 diabetes. Given the same glycemia status, the age- and sex-adjusted prevalence of decreased eGFR was much lower than that of albuminuria.

Table 2 shows odds ratios (ORs) and their 95% confidence intervals (CIs) of decreased eGFR and albuminuria for age and gender, glycemic status, lifestyle behaviors, and components of metabolic syndrome as they are entered into the model sequentially. Without any adjustment, age was significantly associated with decreased eGFR. Type 2 diabetes was significantly associated with decreased eGFR with adjustment of age and gender. After additionally adjusting to lifestyle behaviors and the other components of metabolic syndrome, the OR decreased slightly and was still statistically significant (OR: 2.05, 95% CI: 1.18–3.58). The other factors that were significantly associated with decreased eGFR were age and high triglyceride level. For albuminuria, age was significantly associated with albuminuria. When type 2 diabetes and hyperglycemia were entered, only type 2 diabetes was significantly associated with albuminuria. After additionally adjusting to lifestyle behaviors and the other components of metabolic syndrome, the OR for type 2 diabetes was still statistically significant (OR: 2.93, 2.11–4.07). The other factors that were significantly associated with albuminuria were age, higher waist circumference, high triglyceride, and high blood pressure. The findings of sensitivity analysis show that the ORs of decreased eGFR and albuminuria for type 2 diabetes were similar (multivariate-adjusted OR: 2.93, 1.12–7.67 for decreased eGFR; 2.34, 1.72–3.19 for albuminuria).

## 4. Discussion

Using a representative random sample of a Taiwanese metropolitan population, our study first identified a strong, positive, and significant relationship between type 2 diabetes and decreased eGFR and albuminuria, which is a unique epidemiological finding. The prevalence of decreased eGFR and albuminuria increased as the glycemic level increased. These relationships were independent of age, sex, smoking, drinking, high triglyceride, high waist circumference, high blood pressure, and low HDL-C. The ORs of albuminuria for type 2 diabetes had a higher precision. On the other hand, the values of the ORs of decreased eGFR for type 2 diabetes had larger variation with lower precision.

Previous epidemiologic studies have demonstrated that diabetes is one of the major risk factors for the development and progression of chronic kidney disease and microalbuminuria [22, 23]. Some cross-sectional studies also have examined the association between hyperglycemia and chronic kidney disease and/or microalbuminuria [6–9, 11, 24, 25]. A cross-sectional survey of 6,217 residents aged 20 years or older in the US general population found that hyperglycemia was not associated with decreased eGFR but that they had 2.53-fold higher odds of microalbuminuria than persons with no traits [11]. Palaniappan and colleagues examined the relationship between hyperglycemia and microalbuminuria only and did not examine the relationship between hyperglycemia and eGFR. They found that hyperglycemia demonstrated a strong association with microalbuminuria in both men and women, with ORs of 2.24 and 2.51, respectively [24].

Our data indicated that high triglyceride was the only component of metabolic syndrome that was associated with decreased eGFR, in addition to glycemic status. On the other hand, all components of metabolic syndrome were associated with albuminuria. These findings may indicate that our study had a limited ability to detect the relationships between decreased eGFR and high waist circumference, high blood pressure, and low HDL, due to the low prevalence of decreased eGFR. The other possible explanation was that decreased eGFR and albuminuria were associated with different metabolic syndrome components. If the latter was true, it would imply that different mechanisms played a role in the pathology of decreased renal function in the early and late stages. In addition, we may need different public health strategies for the prevention of albuminuria and decreased eGFR.

We did not observe a significantly increasing prevalence of decreased eGFR and albuminuria in individuals with hyperglycemia. One possible explanation is that individuals with hyperglycemia have hyperfiltration in the early stages of diabetes [21]. In studies of the natural progression of diabetic nephropathy in Pima Indians, individuals with hyperglycemia and newly diagnosed diabetes had higher mean GFRs at baseline than individuals with normal glycemia.

Hyperglycemia, high blood pressure, hyperlipidemia, and abdominal obesity are related to the development of new-onset kidney disease [25, 26]. There are several potential mechanisms underlying the effects of these factors. Hyperinsulinemia may have a significant role in renal dysfunction. Triglyceride-rich apolipoprotein B-containing lipoproteins may promote the progression of renal insufficiency [27]. Obesity is related to hyperlipidemia, hypertension, and type 2 diabetes. As the prevalence of obesity increases, obesity-related glomerulopathy becomes an increasingly prevalent condition.

The existence of urinary albumin is a cardinal feature of kidney disease in general. The importance of microalbuminuria has been recognized, especially its association with an increased incidence of cardiovascular disease and events and with the progression of chronic kidney disease. The toxicity of microalbuminuria to the nephron unit results in inflammation and progressive dysfunction of nephron loss [28]. Angiotensin receptor blocker for hypertension and statin therapy for hyperlipidemia might suppress the pathways to renal injury and abrogate the deleterious changes in the glomerulus. Therefore, with early intervention, it is likely that the natural progression of chronic kidney disease and diabetic nephropathy can be delayed. With the high prevalence of albuminuria in this community random sample, screening for albuminuria is needed.

The strengths of our study include the use of a population-based sample not selected for type 2 diabetes, decreased renal function, or cardiovascular disease risk factors and standardized measurements for exposures and outcomes. Potential limitations of our study should be noted. First, the cross-sectional study design makes it difficult to infer causality between type 2 diabetes and the risk of decreased eGFR or albuminuria. Second, eGFR based on serum creatinine levels was used to define chronic kidney disease in our study. Although eGFR is not as sensitive as insulin or iothalamate clearance techniques, it has been used widely in large epidemiologic studies and in clinical practice for estimating renal function. In particular, we adopted the MDRD study equation modified for ethnic Chinese. Thus, our findings are applicable to clinical and public health practice settings. Third, only one morning urine sample was obtained for UACR measurement, which is commonly done in epidemiologic studies. There is potential measurement error due to albuminuria variability and false positive results [29].

In conclusion, we documented that type 2 diabetes is a strong and independent risk factor for decreased eGFR and albuminuria. The stratification risks of albuminuria and decreased eGFR for glycemic status according to metabolic syndrome components were provided. Our findings warrant intervention studies to test the effect of treating type 2 diabetes on reducing the risk of decreased eGFR and albuminuria.

## Figures and Tables

**Table 1:** Demographic factors and cardiovascular risk factors of subjects with renal dysfunction.

Variables	eGFR (mL/min/1.73 m^2^)		*P* value	Albuminuria (mg g^−1^)		*P* value
	≥60 (*N* = 2272)	<60 (*N* = 87)		≤30 (*N* = 2045)	>30 (*N* = 305)	
*Demographic factors *						
Age (years)^†^	56.31 (11.30)	71.03 (9.37)	<0.001	55.86 (11.18)	63.33 (12.05)	<0.001
Age			<0.001			<0.001
<65 (years)	1518 (66.81)	9 (10.34)		1394 (68.17)	129 (42.30)	
≥65 (years)	754 (33.19)	78 (89.66)		651 (31.83)	176 (57.70)	
Sex			0.008			0.13
Male	1092 (48.06)	55 (63.22)		981 (47.97)	161 (52.79)	
Female	1180 (51.94)	32 (36.78)		1064 (52.03)	144 (47.21)	
*Life style behaviors *						
Smoking	361 (15.90)	12 (13.95)	0.74	317 (15.52)	54 (17.76)	0.36
Drinking	530 (23.34)	13 (15.12)	0.10	473 (23.15)	68 (22.30)	0.80
Betel nut chewing	82 (3.62)	1 (1.18)	0.37	74 (3.63)	9 (2.96)	0.67
Exercise	1529 (67.33)	56 (65.12)	0.76	1376 (67.35)	204 (66.89)	0.92
*Diabetes-related variables *						
Family history of diabetes	554 (24.39)	9 (10.34)	0.004	489 (23.91)	71 (23.36)	0.89
Antihypertension medication use	441 (19.41)	50 (57.47)	<0.001	361 (17.65)	126 (41.31)	<0.001
Cholesterol lowering medication use	113 (4.97)	18 (20.69)	<0.001	97 (4.74)	34 (11.15)	<0.001
*Clinical indexes *						
Peripheral vascular disease	140 (6.16)	12 (13.79)	0.009	130 (6.36)	21 (6.89)	0.82
Arterial stiffness	1472 (65.31)	83 (95.40)	<0.001	1271 (62.64)	276 (91.09)	<0.001
Higher Framingham risk scores	801 (35.26)	57 (65.52)	<0.001	661 (32.32)	193 (63.28)	<0.001
BMI ≥ 24 (kg/m^2^)	1146 (50.44)	51 (58.62)	0.17	1020 (49.88)	174 (57.05)	0.03
Diastolic blood pressure^†^	78.74 (12.34)	83.94 (13.81)	<0.001	77.94 (11.92)	85.51 (13.76)	<0.001
Systolic blood pressure^†^	135.00 (21.54)	153.90 (25.97)	<0.001	133.10 (20.39)	152.60 (24.58)	<0.001
Albuminuria	261 (11.51)	44 (53.66)	<0.001	—	—	—
eGFR < 60 (mL/min/1.73 m^2^)	—	—	—	38 (1.86)	44 (14.43)	<0.001
*Metabolic syndrome components *						
High blood sugar	831 (36.58)	51 (58.62)	<0.001	704 (34.43)	173 (56.72)	<0.001
High triglyceride	605 (26.63)	41 (47.13)	<0.001	515 (25.18)	131 (42.95)	<0.001
High waist	651 (28.65)	35 (40.23)	0.03	549 (26.85)	134 (43.93)	<0.001
High blood pressure	1343 (59.11)	77 (88.51)	<0.001	1151 (56.48)	258 (84.59)	<0.001
Low HDL cholesterol	1243 (54.71)	60 (68.97)	0.01	1094 (53.50)	203 (66.56)	<0.001
*Metabolic syndrome *	851 (37.46)	56 (64.37)	<0.001	713 (34.87)	189 (61.97)	<0.001

^†^Mean ± SD; albuminuria: albumin-creatinine ratio (ACR) >30 mg g^−1^ creatinine; peripheral vascular disease (PVD): ankle-brachial index (ABI) <0.9; arterial stiffness: brachial-ankle pulse wave velocity (baPWV) >1400 cm/s; Framingham risk score greater than the cutoff value of 3rd quartile (≥9).

**Table 2:** Odds ratios and their 95% confidence intervals of decreased eGFR and albuminuria for glycemic status from hierarchical logistic regression models.

Variables	Decreased eGFR OR (95% CI)				Albuminuria OR (95% CI)			
	Model 1	Model 2	Model 3	Model 4	Model 1	Model 2	Model 3	Model 4
Age (per year)	1.11*** (1.08–1.13)	1.10*** (1.08–1.12)	1.10*** (1.08–1.12)	1.10*** (1.07–1.12)	1.05*** (1.04–1.06)	1.05*** (1.04–1.06)	1.05*** (1.04–1.06)	1.04*** (1.03–1.05)
Sex (men as reference)	0.83 (0.52–1.32)	0.82 (0.51–1.31)	0.81 (0.49–1.34)	0.79 (0.48–1.32)	1.01 (0.79–1.30)	1.05 (0.81–1.36)	1.19 (0.90–1.59)	1.21 (0.91–1.62)

Hyperglycemia (normal glycemia as reference)		0.99 (0.56–1.78)	1.02 (0.57–1.83)	0.90 (0.49–1.62)		1.28 (0.94–1.74)	1.28 (0.94–1.74)	1.11 (0.81–1.53)
DM (normal glycemia as reference)		2.82*** (1.67–4.76)	2.82*** (1.67–4.76)	2.05* (1.18–3.58)		3.93*** (2.87–5.39)	3.96*** (2.89–5.43)	2.93*** (2.11–4.07)

Smoking			1.27 (0.64–2.53)	1.17 (0.58–2.35)			1.51* (1.05–2.18)	1.38 (0.95–2.01)
Drinking			0.79 (0.41–1.52)	0.80 (0.41–1.57)			1.12 (0.80–1.56)	1.11 (0.79–1.55)

High triglyceride (<150 mg/dL as reference)				1.86* (1.12–3.08)				1.34* (1.02–1.76)
High waist (men ≤ 90 cm, women ≤ 80 cm as reference)				1.03 (0.63–1.66)				1.40* (1.05–1.87)
High blood pressure (<130/85 mmHg as reference)				1.63 (0.79–3.37)				2.19*** (1.53–3.12)
Low HDL cholesterol (men ≥ 40 mg/dL, women ≥ 50 mg/dL as reference)				1.29 (0.75–2.21)				1.21 (0.91–1.61)

*P < 0.05; ***P < 0.001.
